# Poison of Lead Pipes

**Published:** 1872-04

**Authors:** 


### POISON OF LEAD PIPES.

Scientific research has at length determined the following practical facts: —

First, For at least one month after the pipes are laid, no water from them should be used for purposes of drinking and cooking.

Second, If the water contains lime (carbonate) to the extent of four grains in a gallon, the pipes are not acted upon by water passing through lead pipes.

To know the quantity of lime contained in water, boil a gallon until it has disappeared, and weigh the remnant. If you blow through a quill or rye straw into a glass of water, it becomes of a milky color if there is lime in it.

If the water is soft, it forms on the inside of the lead pipes a coating, called the oxide of lead, which coating more effectually prevents the decomposition of the lead beyond it than a coating of tin or zinc, and costs nothing.

